# Searching for intra-cloud positive leaders in VHF

**DOI:** 10.1038/s41598-023-41218-x

**Published:** 2023-09-02

**Authors:** O. Scholten, B. M. Hare, J. Dwyer, N. Liu, C. Sterpka, K. Mulrey, S. Ter Veen

**Affiliations:** 1 University Groningen, Kapteyn Astronomical Institute, Landleven 12, 9747 AD Groningen, The Netherlands; 2 Interuniversity Institute for High-Energy, Vrije Universiteit Brussel, Pleinlaan 2, 1050 Brussels, Belgium; 3 Netherlands Institute of Radio Astronomy (ASTRON), Postbus 2, 7990 AA Dwingeloo, The Netherlands; 4 Department of Physics & Astronomy and Space Science Center (EOS), University of New Hampshire, NH Durham, 03824 USA; 5 Department of Astrophysics/IMAPP, Radboud University Nijmegen, P.O. Box 9010, 6500 GL Nijmegen, The Netherlands

**Keywords:** Natural hazards, Physics

## Abstract

We have used the LOw-Frequency ARray (LOFAR) to search for the growing tip of an intra-cloud (IC) positive leader. Even with our most sensitive beamforming method, where we coherently add the signals of about 170 antenna pairs, we were not able to detect any emission from the tip. Instead, we put constraints on the emissivity of very-high frequency (VHF) radiation from the tip at 0.5 pJ/MHz at 60 MHz, integrated over 100 ns. The limit is independent on whether this emission is in the form of short pulses or continuously radiating. The non-observation of VHF radiation from intra-cloud positive leaders implies that they proceed in an extremely gradual process, which is in sharp contrast with the observations of other parts of a lightning discharge.

## Introduction

The detection of emitted VHF radio waves is an efficient method to image the development of a lightning discharge since VHF is virtually not attenuated in the atmosphere and thus not obscured by clouds. This thus allows for studying the complete development of the discharge. While VHF is very efficient in detecting negative discharges, the detection of positive discharges has proven to be much more difficult. Except for a few cases such as when positive leaders that propagate to ground^1–3^, are emitted from a tall building^2,4^, or occur in rocket-triggered lightning^5^ they elude direct detection by VHF. If at all, inter and intra cloud positive leaders show in VHF-images only indirectly through small scale discharges, such as needles, along their tracks^6–9^ or through dart (sometimes called recoil or K-change) leaders^10^.

From a large number of observations we know that positive leaders show in VHF very differently from negative leaders. The growing tips of negative leaders are clearly visible by the copious amounts of VHF emitted due to their step-wise propagation. Newly formed positive leaders can sometimes be traced by intermittent VHF emission along their track, known as needles^6,7,9^, often followed by a dart leader that may propagate all along the track towards the negative leader at the other end. These intermittent VHF emissions along the track, by their intermittent and random nature, cannot be the growing tip of the positive leader.

In this work we search for the growing tip of the positive leader. Since, at any time, the positive leader tip must be farther along the channel than the currently observed VHF emission, we search along the positive leader track before the time the track becomes observable by the needle activity (intermittent VHF emission) or by a dart leader. How much earlier is, however, not known and is an issue of much interest. To do so we have performed a specific search for this tip using the LOw-Frequency ARray (LOFAR)^11^ which, by coherently adding the signals of about 170 antenna pairs, offers the most sensitive VHF based lightning-imaging method to date.

We concentrate on one particular section of a rather long positive leader track where we search for evidence of the growing tip all along the track in a few different time windows. These time periods are selected to have a minimum activity elsewhere in the discharge thus to be able to achieve the highest possible sensitivity. The flash in this study is one of very few LOFAR observed flashes that shows VHF-quiet time periods during its development. Assuming the positive leaders propagate continuously, the VHF-quiet periods provide an opportunity for LOFAR to map any radiation sources at the tips of the positive leaders. In spite of the high sensitivity we have not been able to locate the tip and have, instead, set limits on its VHF brightness.

Our lack of observation of the positive leader tip is rather surprising. Leader tips carry a considerable electric charge in the form of a very dynamic streamer corona. Any accelerating charge will emit electromagnetic radiation where the emission is most intense when the time scale of the acceleration is commeasuring with the oscillation frequency of the radiation. Thus it can be understood why negative leaders are very VHF bright. The non-observation of VHF from the positive leaders thus leads to the conclusion that they propagate in a very gradual, continuous mode, completely contrary to the stepping process seen for negative leaders. In a lightning discharge thus two, totally different, propagation modes appear simultaneously where the negative end is proceeding in an extremely VHF-bright stepping mode while the other, electrically connected, positive end is proceeding in an extremely VHF-quiet continuous mode. It is not clear how this can be reconciled in one consistent model. The observation of the VHF-quiet mode for positive leaders in lightning appear to be consistent with the observations for large-gap sparks^12^.

In "Data" Sect. we discuss the lightning flash, data, and imaging procedures used. In "Result" Sect. we elaborate on the procedure used to demonstrate we have a non-detection. This includes, first, a discussion of the flash and the positive leader of interest. Followed by a discussion of the statistical approach we used to establish an upper-limit of the intensity. We also discuss a weak radio source that we stumbled upon during this procedure that was not from the positive leader tip. In "Discussion" Sect. we discuss our evidence that we do not see the positive leader tip, and we contrast this with the observations of VHF emission from positive leaders near ground. Finally, we summarize in "Summary" Sect.

## Data

For this work we use a 1.5 second LOFAR recording from lightning flashes that occurred on April 24, 2019 at 19:44:32 UTC, which we label as 19A-1. An complete overview of the flashes in this recording is shown in the Supporting Information.

LOFAR^11^ is a radio telescope consisting of several thousands antennas spread over much of Europe. For these observations we record the signals of antennas in selected Dutch stations only. For the present work we use about 170 dual-polarized antennas spread over 34 clusters (called stations), operating in the 30 – 80 MHz VHF-band with base lines up to 100 km. To build images from the observations we use two different procedures. One is using the impulsive imager, described in^13^, where a time-of-arrival-difference method is used to locate the sources of the VHF pulses over the full extent of the discharge. Time of arrival differences are calculated using interferometric cross correlations. In the second imaging method, called TRI-D^14,15^, the volume to be imaged is voxelated where for each voxel the beam-formed intensity is calculated for 100 ns sections of the time trace. For beamforming, a coherent sum over all antenna traces is made while keeping track of their polarization.

With the TRI-D imager a very complete picture is obtained for all, including weak, sources in a limited volume. With the TRI-D imager it is possible to image weak sources with a sensitivity that reaches below the natural background^16^, due to the galactic background radiation, even for cases where the sources do not produce distinct pulses. The impulsive imager, in contrast, obtains the location of sources for which the emitted pulses can be identified in (almost) all antennas. Both imaging methods use the signals of all recorded antennas.

**Figure 1:**
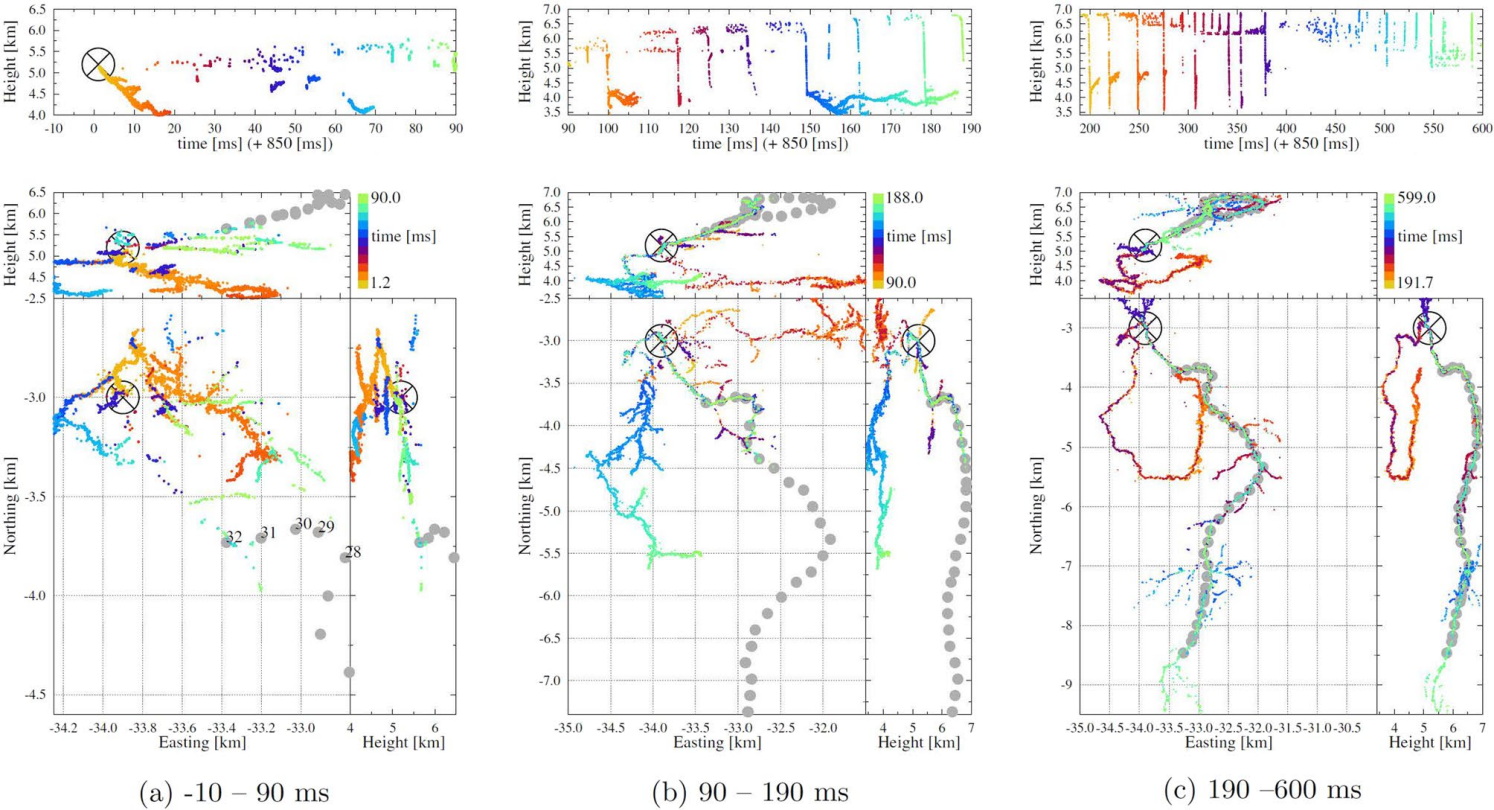
An overview of a series of dart leaders along the track of the positive leader on flash 19A-1 that are of interest for this work. The sub figures show successive time periods, sub figure (**a**) shows the beginning of the flash to t=90 ms, sub figure (**b**) the following period until t=190 ms while sub figure (**c**) shows the later part up to t=600 ms when the last dart leaders passes through the section of interest, where its track was used to mark the positive leaders track. The gray circles indicate the track of the inferred positive leader that is investigated in this work where on the left the numbering is given as used later in this work. The $\bigotimes$ mark indicates the initiation of the flash.

Figure 1 shows subsequent time periods of a section of flash 19A-1 that is the focus for this work. The flash initiated close to (−3.0, −33.9, 5.2) km (indicated by a $\bigotimes$ in Fig. 1) at t=0 ms. Several negative leaders are seen to propagate out and downward from the initiation point to altitude below 4.5 km. This negative leader propagation occured with starts and stops and can be seen in all three panels of Fig. 1. In the meantime, an inferred positive leader propagated upward, as indicated by the gray circles in Fig. 1. The early part of the track of the positive leader is visible in Fig. 1a-top panel by the scattered sources at altitudes above 5 km as well as some rather short dart leaders. In Fig. 1b the dart leaders are more visible as the discharge is evolving. Some of these dart leaders, such as the ones around t=100 and 150 ms, start in the positive leader and, once they reach the end of the negative leader, trigger the further propagation of this negative leader. As seen in Fig. 1c, the dart leaders until t=280 ms initiated negative leaders that curl (in the ground-plane projection) under the track of the positive leader. The later ones feed a negative leader complex north of the initiation site that has been truncated in order to focus on the section of present interest. The track of the positive leader is most clearly visible from the multiple dart leaders at t=530 to 600 ms. It is seen that the positive leader stretches a long distance south and is still visible in the most southern imaged area of Fig. 1c. The part of the positive leader track that is investigated in this work is indicated by the string of grey dots and is obtained from the track of the dart leader at t=600 ms, but all dart leaders pass through the same track. The positions of the dots mark the centers of the image cubes where bordering ones just overlap as to give a continuous coverage of the whole track.

## Results

To search for the growing tip of the positive leaders we use the TRI-D imager since it, by employing beamforming techniques, is able to locate much weaker sources than the impulsive imager and also sources that emit more continuously. The best sensitivity is reached when the VHF background, due to strong VHF sources elsewhere in the flash, is minimal. For flash 19A-1 we selected a few VHF-quiet time periods, where most periods fall between times 70 and 83 ms (when needle activity is seen at the base of the positive leader, see Fig. 1a) and two for times around 138.0 ms (when needle activity starts to become visible along the grey-dotted track in Fig. 1b). For each of the selected time periods we use the TRI-D imager for 0.3 ms of data to image the possible sources in a sequence of image cubes arranged along the track of the positive leader where no earlier activity was observed and thus where we should expect to find the tip of the positive leader.

To set a limit on the distance the growing tip may have moved from the region where the needle activity is seen, we estimate the propagation velocity of the positive leader. From Fig. 1a it can be seen that in the first 90 ms the VHF-visible portion of the positive leader (i.e. the needles) has grown to a distance of about 1.5 km from the initiation point of the flash indicated by the $\bigotimes$ in Fig. 1. From the middle panel it can be seen that in the following 100 ms the needles have appeared along an additional 2 km. Thus, the needles appear along the positive leader channel at a speed of the order of $2\times 10^4$ m/s which is very similar to the estimates found in the literature, see^7,9,17^.

On the basis of the estimated speed it seems reasonable to expect that the tip of the positive leader is not more distant from the needle activity than the distance it can travel in 100 ms, thus 2 km. To be conservative, we have searched for the tip over a distance of 5 km along the dart leader shown in Fig. 1, where the searched track is indicated by the grey dots. In fact, each grey dot marks the center of an image cube of size (200, 180, 250) m located such that their borders just overlap. The size of each voxel is $2\times 1.5\times 5$ m, a compromise between having a good resolution and having a manageable number of voxels, where the position of a source in interpolated between different voxels, see^14^. The relative size of a voxels is reflecting the point-spread function of the system in the three directions. For each image cube the beam-formed intensity is calculated for the $(100\times 120\times 50)=6\times 10^6$ voxels of the image cube for all of the 3000 time slices of 100 ns in a 0.3 ms interval centered at a few different times. The first time interval is taken 5 ms before initiation of this flash to learn about the intensity of the background. Ten intervals are taken between 70 and 84 ms, and two around 138 ms after the start of the flash. The image cubes closest to the initiation point of this flash are placed at the position where needle activity is seen at t=70 till 85 ms.

**Figure 2:**
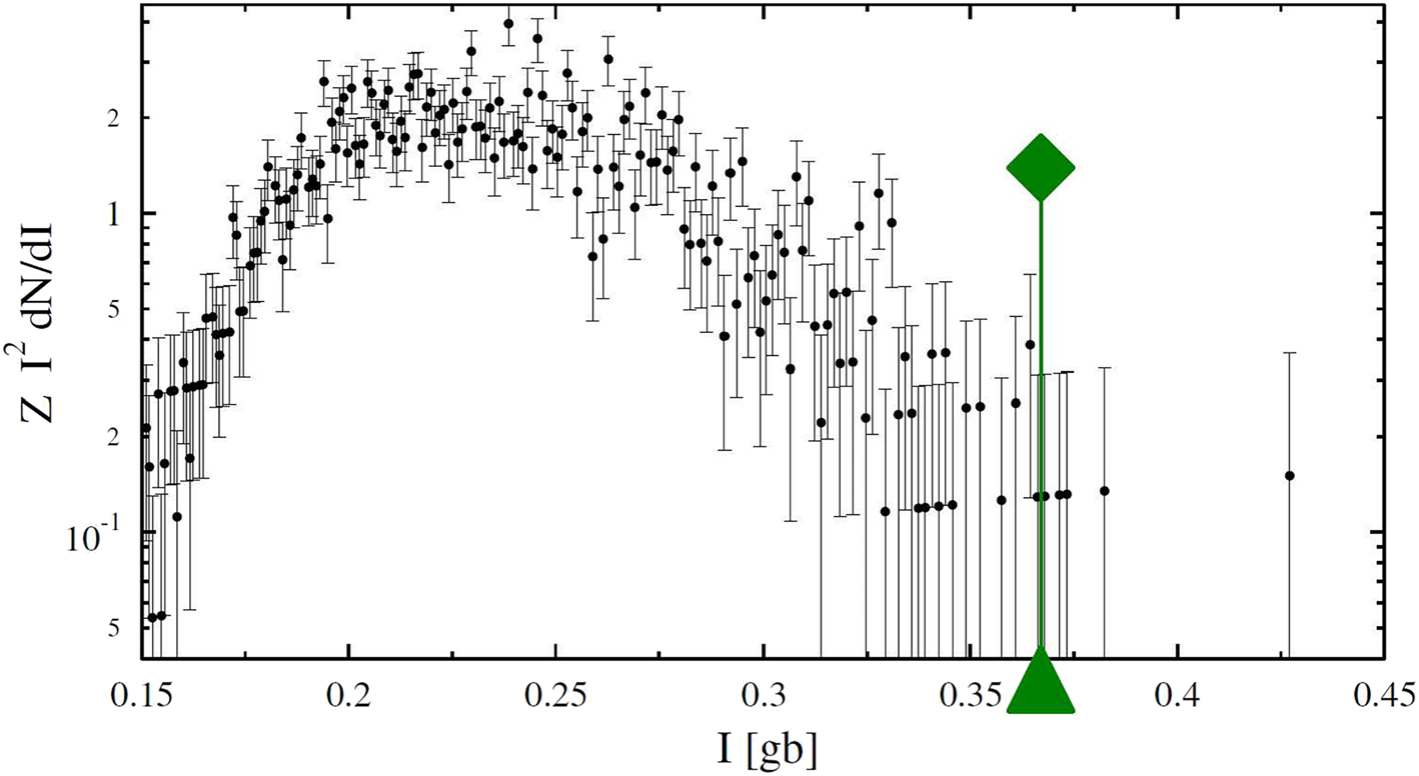
A typical plot showing the normalized number density of sources (multiplied by $I^2$) as function of the intensity, *I* (in units [gb], see text) for one image cube over a time span of 0.3 ms. The vertical axis is scaled by $I^2$ to compensate a generic decrease in source density with intensity. The green line marks the $I_5$ value (see text) for this tesseract.

In each image cube, following the common procedure for the TRI-D imager, the voxel with maximal intensity is found for every time slice of 100 ns. This is used for finding the source location following an interpolation procedure with the neighboring voxels. Each image cube thus yields 3000 source locations and source intensities (at most, since sources at the borders are excluded). A typical intensity distribution for an image tesseract (3-D cube and time) is shown in Fig. 2. It was shown in an earlier study that the intensity spectrum resembles a power law^18^ and, to compensate the strong fall-off at higher intensities, the spectrum is multiplied by $I^2$. A normalization factor $Z^{-1} = \int I dN$ is introduced to make the units dimensionless. Since the total number of sources in an image tesseract is limited by having at most one source per 100 ns, the number of sources must have a cutoff at smaller amplitudes, which is seen very clearly in Fig. 2. To have a quantitative measure of source strengths we introduce $I_N$, the intensity for which there are *N* sources that have an intensity exceeding $I_N$ for a specific tesseract. In Fig. 2 a green marker is placed at the strength $I_5=0.3670$ gb thus marking the point where there are only 5 sources found with a larger intensity.

The intensity of the sources as determined by the TRI-D imager is usually expressed in units of [gb]^14,16^ (normalized to the galactic background noise, see Supporting Information for a more extensive discussion of this unit). A source with strength *I* gb emits at a spectral energy density, integrated over the full solid angle and over a time slice $\Delta _t$ (fixed to $\Delta _t$=100 ns in this work), of

1 $$\begin{aligned} F=11 \times \frac{I}{\textrm{gb}} \frac{\Delta _t}{100\;\textrm{ns}} \; \mathrm{pJ/MHz} . \end{aligned}$$

To obtain this relation we placed a simulated dipole source with strength of I gb at an altitude of *D* km vertically above an antenna. The deposited and energy in the antenna can be expressed in terms of the background power over the slice duration, where the power in the background is determined from the temperature of the galactic background while adding instrumental noise^19^.

**Figure 3:**
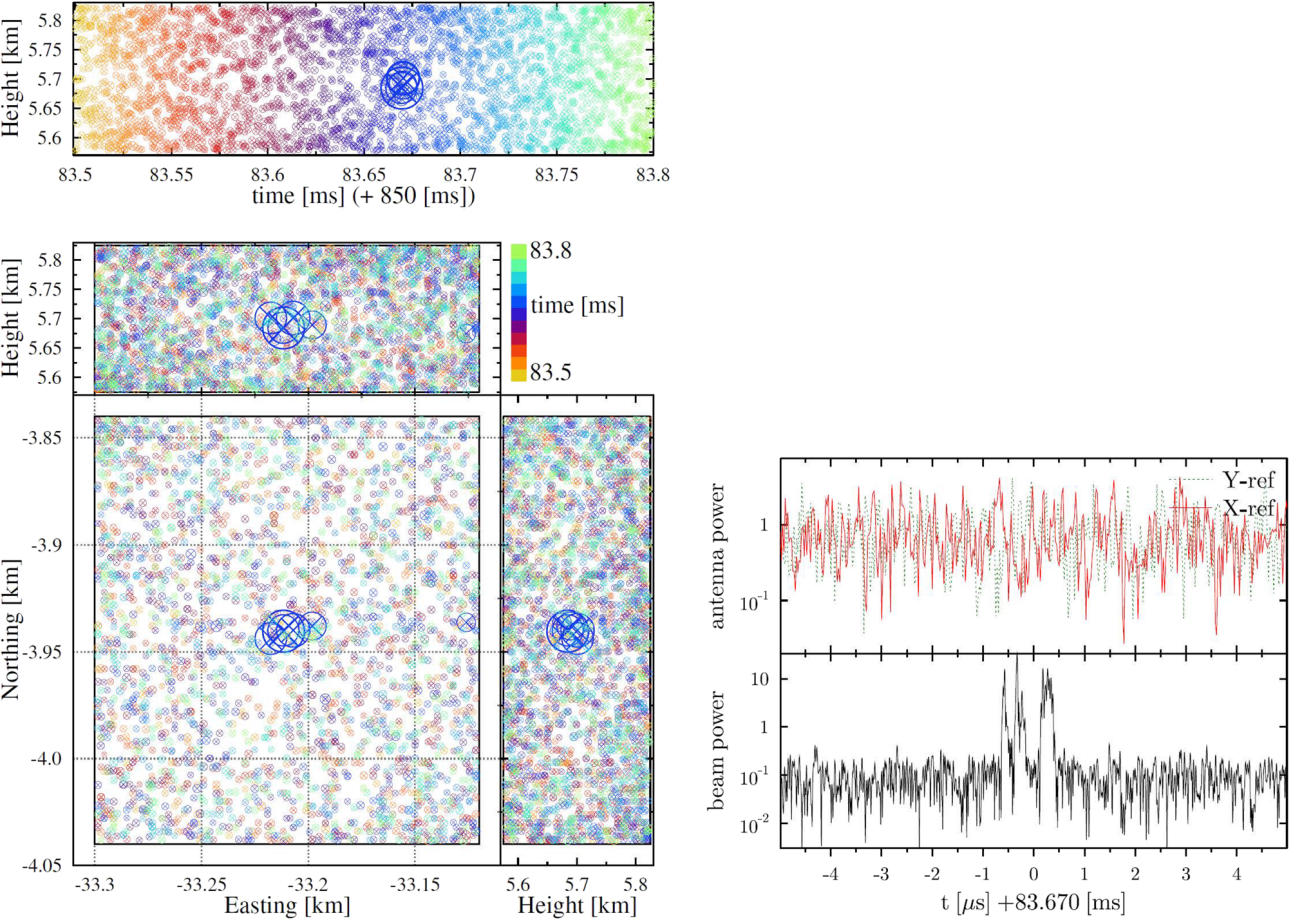
The source that is excluded from the t=83.6 ms time bin. The left shows the TRI-D image made using an image cube of the same size as in the other analyzes presented in this work. The size of the circles is proportional to the intensity. On the right the power spectrum for an antenna pair in the core of LOFAR is shown in the top panel while the bottom panel shows the beamed spectrum where the signals of 170 antenna-pair have been added coherently for the voxel at the center of the image cube shown to the left where, for display purposes, only 10 μs of the full 0.3 ms spectrum is shown. For each voxel such a spectrum is build.

In performing imaging in the 0.3 ms time interval around t=83.6 ms we noted an increased intensity for the time span 83.665–83.675, simultaneously in many different tesseracts, thus excluding it being a genuine source in one of the image cubes. Close investigation revealed that this was due to side beams from a genuine source in the vicinity of the image cubes that was not picked-up by the impulsive imager. This time span was thus excluded from the further search. With the TRI-D imager we succeeded to determine the precise location of this source at (N, E, h)=(−3.94, −33.21, 5.7 ) km as shown in Fig. 3-left with an intensity of about 10 gb. Interesting to note is that this location is the same as that of another source imaged by the impulsive imager at t=81.8 ms, residing on a side branch of the positive leader. The multi-colored background in Fig. 3-left is due to noise sources that are evenly distributed over the image tesseract with an intensity distribution similar to what is shown in Fig. 2. To be able to appreciate the sensitivity of the TRI-D imager the top panel on the right of Fig. 3 shows the power time trace (square of the amplitude) for the two signals of an antenna pair from the LOFAR core while the bottom shows the coherent sum for the center of the image cube on the left. While in the core antenna there is hardly any evidence for a pulse it shows in the TRI-D image with an intensity of about 10 gb. The signal power received by an antenna from such a source is determined by the angle dependent gain of the antenna and inversely proportional to the square of the distance of the source to the antenna while the noise background is (almost) constant. A source of 20 gb at 33 km distance is thus hardly visible in the time trace because the antenna gain for a source at a zenith angle of 80° is small. A more extensive discussion is presented in the Supporting Information.

As a signature of the presence of the growing tip of the positive leader we look for excess intensity in the different image tesseracts along the positive leader as compared to what was found as background, i.e. the intensity at 5 ms before the start of the flash. To quantify the intensity we have opted for using $I_N$ with $N=5$. On the one hand we want *N* to be small in order to be sensitive to less frequent peaks in VHF emission from the tip and, at the other, not too small to limit the influence of statistical fluctuations. If the tip of the positive leader would glow (in VHF) constantly with an intensity of $I_{\textrm{tip}}$ (over each interval of 100 ns in the imaged time interval of 0.3 ms), the intensity spectrum of the sources in the image tesseract containing the tip should show a clear peak at $I_{\textrm{tip}}$ instead of a distribution like shown in Fig. 2, even if $I_{\textrm{tip}}$ were an order of magnitude weaker than the source in Fig. 3. In case that $I_{\textrm{tip}}$ would lie within the bulk of the noise intensities the presence of the tip, by chance constructive interference with the noise, should increase the high-intensity tail shown in Fig. 2 and thus give rise to a larger $I_5$ for the tesseract containing the tip as compared to the other tesseracts. These considerations hold even when the growing tip tends to be diffusely emitting over length scales of tens of meters.

**Figure 4:**
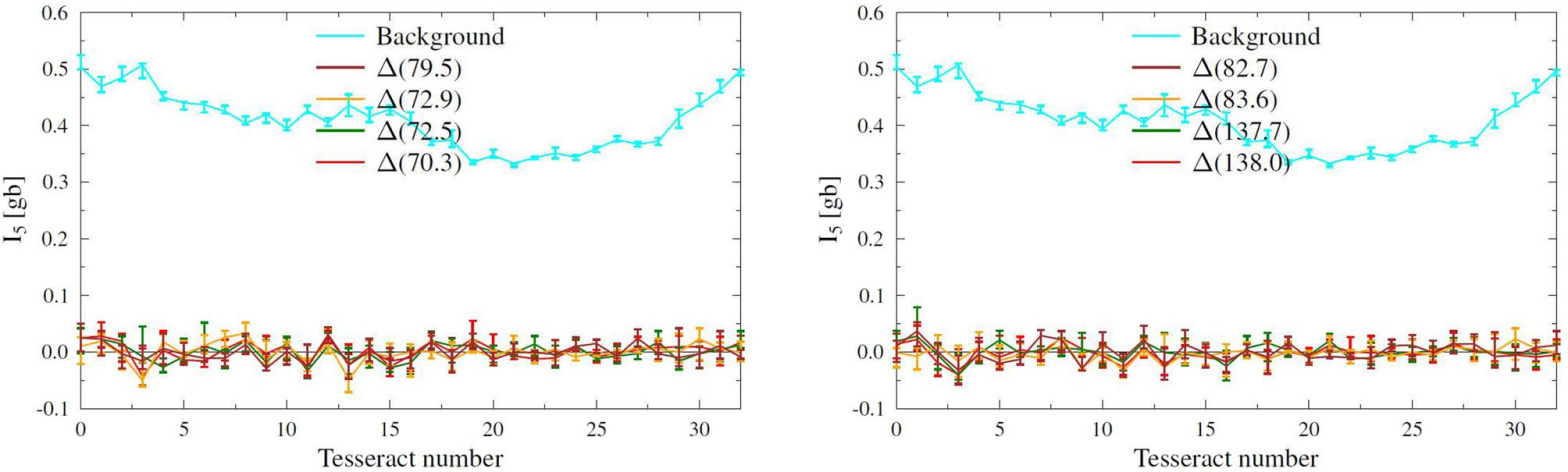
The curve labeled ’Background’ gives the $I_5$ intensities (in [gb]) for successive tesseracts (image cubes calculated for a time-span of 0.3 ms sliced in 100 ns bins) along the track of the positive leader centered around at 5 ms before the flash initiated. The other curves give the difference of the $I_5$ values for the image cubes at specified times with the background values. The highest tesseract numbers are closest to the base of the positive leader, see Fig. 1a.

The central times for the tesseracts of 0.3 ms duration were chosen such that there is a minimum of sources elsewhere in the discharge that, by virtue of ‘side beams’, would introduce false sources and thus inadvertently increase the $I_5$ values. The 32 different locations of the tesseracts were taken as indicated in Fig. 1 where the one with the highest number is nearest to the initiation point. For the earlier image tesseracts there was needle activity seen at the place of the tesseracts close to the base of the positive leader, but (just) outside the time span covered by the tesseract. This to make sure that the propagating tip should be captured in the analysis. The positions of the tesseracts are fixed to a number of specific locations as the intensities of background-noise sources depends on the location, see Fig. 4. The background values for $I_5$ at each position are determined by analyzing tesseracts at 5 ms before the flash initiated, thus making sure that there is no positive leader present yet. To give an impression of the statistical significance of the values we give ‘error’ bars that range from $I_3$ at the upper to $I_7$ at the lower end. The earlier times (between t=70.3 ms and 83.6 ms, see Fig. 4) were chosen while there was needle activity seen (see Fig. 1-left). The later times t=137.7 and 138.0 ms were chosen soon after a dart leader was initiated in this positive leader, just before needle activity started again (see the middle panels of Fig. 1). To express the significance we present in Fig. 4 the differences, $\Delta (t)=I_5(t)-I_5$(Background) for image tesseracts at the identical locations, marked by the numbers given on the horizontal scale in Fig. 4. The ‘error’ bars on $\Delta (t)$ are the sum of those for $I_5$(Background) and $I_5(t)$. In Figure S3 in Supporting Information the analysis is shown for another four selected time periods.

## Discussion

Fig. 4 shows that the differences in $I_5(t)$ values with the noise floor, $\Delta (t)$, are vanishingly small over a distance of about 5 km from the point where needle activity was seen at times when the positive leader is expected to be propagating. From this it can thus be concluded that either (1) the assumption is wrong and the positive leader is not propagating or that (2) the propagating positive leader is emitting VHF radiation at an intensity that is below our detection limit. We find it highly unlikely that the positive leader has stopped propagating since we see needle activity at the base of the positive leader track around the time periods we have investigated. We thus conclude that the tip of the positive leader is extremely VHF quiet while propagating, to the extent that we find no trace of the tip even when using the TRI-D imager under the most favorable conditions.

To set an upper limit on the intensity of the positive leader tip we have argued that $\Delta (t)$ values should be about as large as the VHF-intensity emitted by the tip, $I_{\textrm{tip}}$. Such an emission should show as peak in Fig. 4 at $I_{\textrm{tip}}$ for the particular tesseract in which the tip is propagating. Additionally, the propagating tip should show as a peak in each curve in Fig. 4, that gradually moves to further distances down the channel for tesseracts at later time periods. Obviously, this is not seen. One thus can put a conservative limit of 0.05 gb on $I_{\textrm{tip}}$ of this positive leader. Using Eq. (1), this leads to the conclusion that the spectral energy density of the tip at 60 MHz is less than $F=0.5$ pJ/MHz over a 100 ns slice. Even if the positive leader was propagating in a stepping mode the derived limit is valid as in refs^1,3^ it is shown that stepped positive leaders show stepping times ranging from 30 μs down to a fraction of 5 μs. A time interval of 0.3 ms will therefore contain many steps and the derived intensity limit thus should also apply to the case that the IC positive leader is propagating in a stepping mode.

Another evidence for the VHF quietness of the positive leader can be taken from Fig. 3. For about 0.15 ms before and after observing the source there is no detected activity anywhere along the track of the positive leader. At exactly the same spot a source was imaged by the impulsive imager at t=82 ms, see figure S4 in Supporting Information. These sources lie on a side branch of the main positive leader that is about 1 km long (at least the part made visible through dart leaders). As a second test we have repeated the same search for the positive leader tip along this short side branch reaching to the same conclusion as for the main branch. The results of this analysis are presented in the supporting information figures S2 and S3. We see this as another evidence that the positive leader is active at these times while the growing tip remains invisible in VHF.

Our observation that the tip of intra- and inter-cloud positive leaders is extremely quiet in VHF is in stark contrast with the observation^3^ of a VHF-bright positive leader in a cloud-to-ground (CG) discharge showing clear stepping very similar to what is seen in optical observations^1,2,4,5,20^ of rocket triggered positive leaders and positive leaders emanating from high buildings. Another stark contrast is that the VHF-bright positive leaders propagate at velocities of order $2\times 10^6$ m/s while the one of this work is probably propagating at a speed of only $2\times 10^4$ m/s. Very interesting in this respect is the observation made in^12^ that when triggered lightning is propagating to higher altitudes there is a growing continuous current on which oscillations are superimposed that are gradually damped.

These observations suggest that when the positive leader is approaching a well conducting surface, or is well connected to a large conducting body (=Earth) the charge at its tip must be large with an electric field in the streamer zone that surpasses some critical value apparently causing the leader to be impulsive and propagate with a large speed.

Under laboratory conditions, as discussed in^17^, the positive leader is weakly luminous and shows continuous propagation with a constant glow of streamers at the tip. Its velocity is of order $2 \times 10^4$ m/s which suffices to keep the electric field at the tip sufficiently high to sustain the ionization processes in a diffuse corona region. These observed values for the propagation speed and the observed continuous propagation, implying a constant, non varying current (and thus hardly any VHF emission), are consistent with our observations.

## Summary

We have performed a search for the tip of a positive cloud-to-cloud leader using the TRI-D imager on data from LOFAR. In the process we have been able to set an upper limit of the VHF-emissivity of the tip at 60 MHz of $F=0.5$ pJ/MHz integrated over 100 ns, or of 5 μW/MHz if emitting continuously.

Our results are consistent with the laboratory observations of a positive leader as discussed in^17^. In the laboratory a smooth propagation of the corona at the tip is seen, creating the positive leader behind it in a continuous motion. In such a smooth and continuous process no (hardly any) VHF will be emitted which is consistent with our upper limit. In addition the propagation velocities reported in^17^, $2 \times 10^4$ m/s, are the same as what is deduced for natural CC positive leaders.

It is quite interesting to find that the negative end of a lightning leader emits copious amounts of VHF radiation while the positive end is VHF quiet to such an extent that even in the most favorable circumstances its position cannot be determined by the most sensitive LOFAR imaging methods.

### Supplementary Information

Supplementary Information.

## Data Availability

The data are available from the LOFAR Long Term Archive (for access see^21^), under the following locations: L703974_D20190424T194432.504Z_stat_R000_tbb.h5 all of them with the same prefix srm://srm.grid.sara.nl/pnfs/grid.sara.nl/data/lofar/ops/TBB/lightning/ and where “stat” should be replaced by the name of the station, CS001, CS002, CS003, CS004, CS005, CS006, CS007, CS011, CS013, CS017, CS021, CS024, CS026, CS030, CS031, CS032, CS101, CS103, RS106, CS201, RS205, RS208, RS210, CS301, CS302, RS305, RS306, RS307, RS310, CS401, RS406, RS407, RS409, CS501, RS503, or RS508. To access this data, please create an account following instructions at ^21^ and follow the instructions for “Staging Transient Buffer Board data”. In particular the utility “wget” should be used as in wgethttps://lofar-download.grid.surfsara.nl/lofigrid/SRMFifoGet.py?surl=location where “location” is the location specified in the above. The datasets used and/or analysed during the current study are available from the corresponding author on reasonable request. The software used for data analysis is available at ^22^. Figures 1 till 4 in this work have been made using the Graphics Layout Engine (GLE) ^23^ plotting package. The data displayed in these figures may be retrieved from ^24^.
