# Supplementary material for: Searching for intra-cloud positive leaders in VHF
Source: Sci Rep. 2023 Sep 2;13:14485. doi: 10.1038/s41598-023-41218-x (PMC10475077; doi:10.1038/s41598-023-41218-x)
Supplement: Supplementary file 1 — Supplementary Information. [file 41598_2023_41218_MOESM1_ESM.pdf]

Supplementary Information for "Searching for intra-cloud positive leaders in VHF"

O. Scholten[1,2,*], B. M. Hare[1,3], J. Dwyer[4], N. Liu[4], C. Sterpka[4], K. Mulrey[5], and S. ter Veen[3]

[1]University Groningen, Kapteyn Astronomical Institute, Landleven 12, 9747 AD Groningen, The Netherlands
[2]Interuniversity Institute for High-Energy, Vrije Universiteit Brussel, Pleinlaan 2, 1050 Brussels, Belgium
[3]Netherlands Institute of Radio Astronomy (ASTRON), Postbus 2, 7990 AA Dwingeloo, The Netherlands
[4]Department of Physics and Space Science Center (EOS), University of New Hampshire, Durham NH 03824 USA
[5]Department of Astrophysics/IMAPP, Radboud University Nijmegen, P.O. Box 9010, 6500 GL Nijmegen, The Netherlands
[*]corresponding author, O.Scholten@rug.nl

ABSTRACT

We have used the LOw-Frequency ARray (LOFAR) to search for the growing tip of an intra-cloud (IC) positive leader. Even with our most sensitive beamforming method, where we coherently add the signals of about 170 antenna pairs, we were not able to detect any emission from the tip. Instead, we put constraints on the emissivity of very-high frequency (VHF) radiation from the tip at 0.5 pJ/MHz at 60 MHz, integrated over 100 ns. The limit is independent on whether this emission is in the form of short pulses or continuously radiating. The non-observation of VHF radiation from intra-cloud positive leaders implies that they proceed in an extremely gradual process, which is in sharp contrast with the observations of other parts of a lightning discharge.

1 Introduction

In Text S1 we present a short discussion on the [gb] units used for source intensity. Figure S1 the complete overview is presented of the flash considered for this work as well as that of a nearby one that overlaps in time. Figure S2 shows the side arm of the main positive leader that was also searched for a positive leader tip. The right side of this figure shows the results of this search. Figure S3 show results of searches during additional time slots for the main positive leader as well as the side arm. Figure S4 shows the detailed needle activity around the time the growing tip of the positive leader is searched for.

2 Text S1.

In the discussion of the power of a source that emits a short pulse, where the pulse width is of the order of the impulse response of the system, we will distinguish the intensity of a pulse as measured by a LOFAR antenna and the emitted spectral energy density by a source, denoted by F in units of [J/MHz]. Since the band width of the LOFAR antennas is limited to about 10 MHz, it is not possible to make a claim about the frequency spectrum of the source and we thus rather give the spectral energy density at the central LOFAR frequency of 60 MHz. The pulse power received by an antenna is falling off with the square of the distance D. In addition the LOFAR antenna sensitivity drops with zenith angle of the source as the antennas are primarily made for astronomical observations. As the source will emit in a dipole pattern, the observed pule strength will also depend on the orientation of the dipole with respect to the receiving antenna. Only through a detailed analysis of the pulse polarization directions and strengths detected in the different LOFAR antennas, as is performed by the TRI-D imager[1], the different factors can be unfolded and the magnitude and direction of the emitting dipole, $\vec{I}_s(t)$ is determined. $S_I = Av(|\vec{I}_s(t)|^2)$ is the time average of the source intensities over a TRI-D time slice of typically $\Delta_t = 100$ ns and is expressed in units of [gb][2,3] using an intrinsic normalization to the noise level in the antennas. The impulse-response time for a LOFAR antenna is shorter than the slice length and thus the full power of an impulsive source, which are the vast majority in a lightning discharge, can be fully contained. The calibration through the antenna noise level offers a relatively simple gain calibration of all 170 antenna pairs in our system since the level of the galactic background is known[4].

To relate [gb] to SI-units [J/MHz] we placed in a simulation a dipole source with strength of I gb at an altitude of D km vertically above an antenna (where the antenna gain is maximal) and oriented transverse to the line of sight. This deposits and energy $\frac{I}{D^2}7.5$ times that of the noise level over the slice duration, defining [gb] in terms of the galactic background. The analysis presented in[4] shows that the noise level in a LOFAR antenna corresponds to a power of 2.2×10^{-14} W/MHz/m^2, accounting for instrumental noise. Combining all factors allows to express the total emitted spectral energy density of a source

with strength I gb as

$$F = 11 \times \frac{I}{\text{gb}} \frac{\Delta_t}{100\ \text{ns}}\ \text{pJ/MHz}\,, \tag{1}$$

integrated over the full solid angle of the emitting source, assuming the source radiates as a dipole. We estimate that the calibration is correct within a factor two. An upper limit to the strength of the source tip of 0.05 gb thus implies an upper limit to the spectral energy density of the tip at 60 MHz of $F = 0.5$ pJ/MHz=0.5×10^{-12} J/MHz if it were impulsive which is equivalent to 5 μW/MHz for a continuous emitter.

3 figures

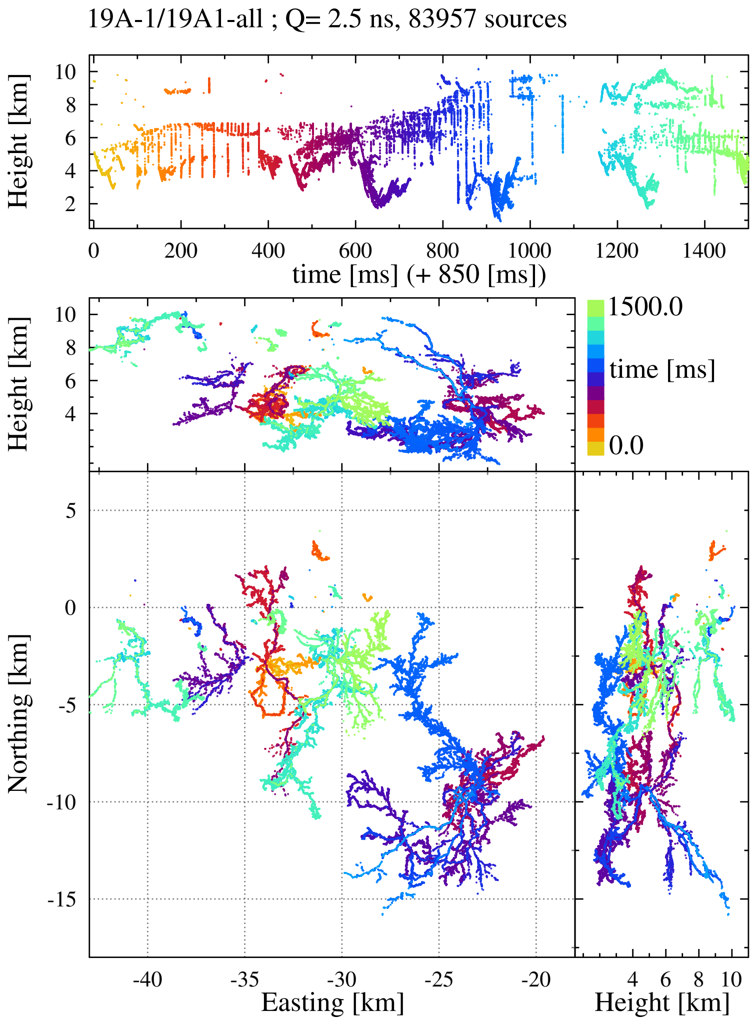

Figure S1. An overview of the flashes for LOFAR recording 19A-1 that occurred on April 24, 2019 at 19:44:32 UTC. The image is made using the impulsive imager showing over 81,500 sources while requiring that the pulse of each source is detected in at least 157 of a total of 172 antennas and that the timing of these pulses is reproduced with an root-means square not larger than 2.5 ns.

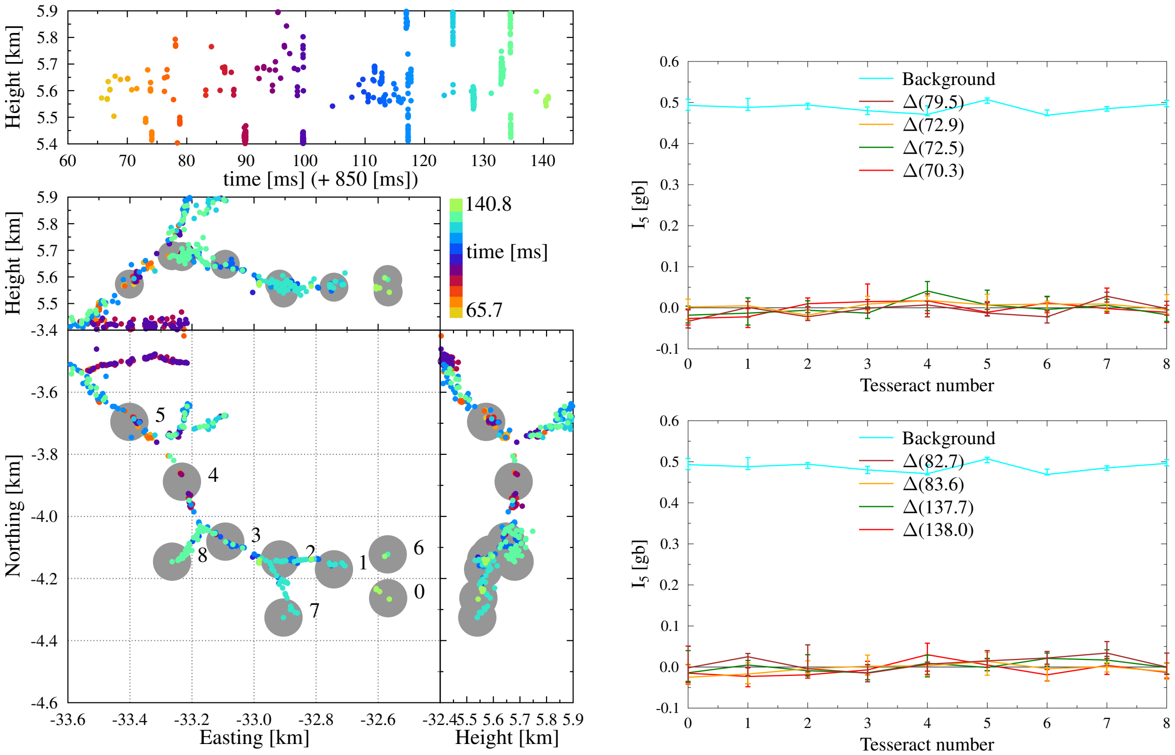

Figure S2. Repeat of the analysis for one of the earlier side branches of the main positive leader.

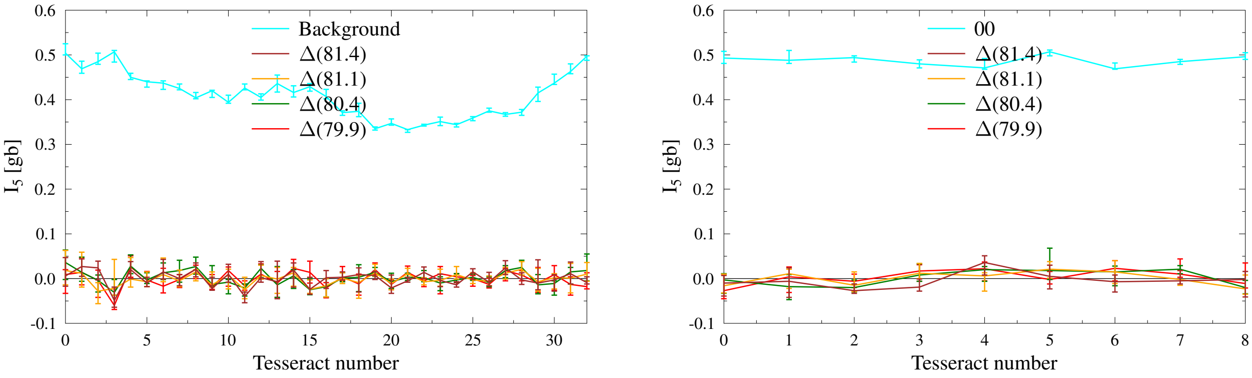

Figure S3. Another Repeat of the analysis for one of the earlier side branches of the main positive leader.

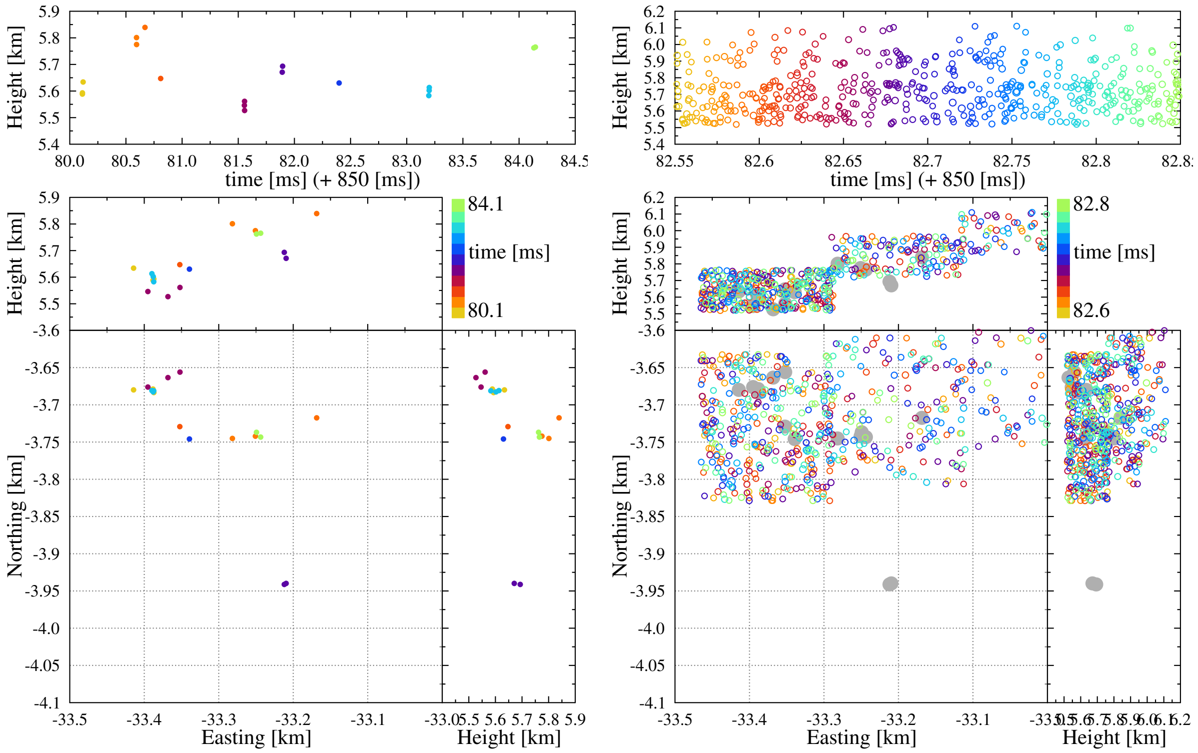

Figure S4. The sources on the end of the positive leader at times when analysis is performed. Right: the nearest image cubes (# 32, 31, and part of 30) for t=82.7 are overlaid. TRI-D sources with an intensity in excess of 0.36 are shown only.

References

1. Scholten, O. *et al.* Interferometric imaging of intensely radiating negative leaders. *Phys. Rev. D* **105**, 062007, DOI: 10.1103/PhysRevD.105.062007 (2022). 2110.02547.
2. Scholten, O. *et al.* Time resolved 3d interferometric imaging of a section of a negative leader with lofar. *Phys. Rev. D* **104**, 063022, DOI: 10.1103/PhysRevD.104.063022 (2021).
3. Sterpka, C. *et al.* The spontaneous nature of lightning initiation revealed. *Geophys. Res. Lett.* **48**, e2021GL095511, DOI: https://doi.org/10.1029/2021GL095511 (2021). E2021GL095511 2021GL095511, https://agupubs.onlinelibrary.wiley.com/doi/pdf/10.1029/2021GL095511.
4. Mulrey, K. *et al.* Calibration of the lofar low-band antennas using the galaxy and a model of the signal chain. *Astropart. Phys.* **111**, 1 – 11, DOI: https://doi.org/10.1016/j.astropartphys.2019.03.004 (2019).
